# Numerical Evaluation of the Influence of Skull Heterogeneity on Transcranial Ultrasonic Focusing

**DOI:** 10.3389/fnins.2020.00317

**Published:** 2020-04-15

**Authors:** Chen Jiang, Dan Li, Feng Xu, Ying Li, Chengcheng Liu, Dean Ta

**Affiliations:** ^1^Department of Electronic Engineering, Fudan University, Shanghai, China; ^2^Institute of Acoustics, Tongji University, Shanghai, China; ^3^State Key Laboratory of ASIC and System, School of Microelectronics, Fudan University, Shanghai, China; ^4^Key Laboratory of Medical Imaging Computing and Computer Assisted Intervention (MICCAI) of Shanghai, Shanghai, China

**Keywords:** transcranial focusing, k-space pseudospectral method, ray tracing, time-reversal theory, skull heterogeneity

## Abstract

In transcranial penetration, ultrasound undergoes refraction, diffraction, multi-reflection, and mode conversion. These factors lead to phase aberration and waveform distortion, which impede the realization of transcranial ultrasonic imaging and therapy. Ray tracing has been used to correct the phase aberration and is computationally more efficient than traditional full-wave simulation. However, when ray tracing has been used for transcranial investigation, it has generally been on the premise that the skull medium is homogeneous. To find suitable homogeneity that balances computational speed and accuracy, the present work investigates how the focus deviates after phase-aberration compensation with ray tracing using time-reversal theory. The waveforms are synthetized with ray tracing for phase aberration, by which the properties of the skull bone are simplified for refraction calculation as those of either (i) the cortical bone or (ii) the mean of the entire skull bone, and the focusing accuracy is evaluated for each hypothesis. The propagation of ultrasound for transcranial focusing is simulated with the elastic model using the k-space pseudospectral method. Unlike the fluid model, the elastic model does not omit shear waves in the skull bones, and the influence of that omission is investigated, with the fluid model resulting in a focal deflection of 0.5 mm. The focusing deviations are huge when the properties of the skull bone are idealized with ray tracing as those of the mean of the entire skull bone. The focusing accuracy improves when the properties of the skull bone are idealized as those of the cortical bone. The results reveal minimal deviation (8.6, 3.9, and 3.2% in the three Cartesian coordinates) in the focal region and suggest that transcranial focusing deflections are caused mostly by ultrasonic refraction on the surface of the skull bone. A heterogeneous skull bone causes wave bending but minimal focusing deflection. The proposed simplification of a homogeneous skull bone is more accurate for transcranial ultrasonic path estimation and offers promising applications in transcranial ultrasonic focusing and imaging.

## Introduction

The transmission of ultrasound through the human cranial bone is very important for non-invasive transcranial acoustic imaging (Errico et al., 2016; Jordan et al., 2017), therapeutic applications such as the ablation of brain tumors (Pernot et al., 2007; Colen and Jolesz, 2010; Mcdannold et al., 2010; Damianou, 2019), and mechanical brain thrombosis ablation angioplasty (Behrens et al., 2001; Liu et al., 2014; Lee et al., 2016; Levinsky et al., 2016). Recently, transcranial ultrasound becomes an alternative approach for neuromodulation techniques, as ultrasound can non-invasively transmit to deep targeted brain circuits (Darrow, 2019; Li et al., 2019). The focusing capability of transcranial ultrasound determines the region and volume of neuron stimulation in deep brain (Tyler, 2011; Ibsen et al., 2015). In addition, it shows capability to detect mental activity based on transcranial acoustic images and functional images (Myrden et al., 2011).

The above applications suggest that transcranial ultrasound can be a promising modality for brain computer interface (BCI) systems. However, the irregular geometrical shape and complicated composition of the cranial bones lead to inevitable distortions in ultrasonic waves, such as propagation-path deflection and phase aberration (Pernot et al., 2003; Kyriakou et al., 2013). Therefore, phase-aberration correction is an important aspect of transcranial ultrasound focusing and imaging.

Over the years, researchers have presented diverse models for studying transcranial ultrasound. For instance, the skull has been idealized as a spherical shell, thereby making it easier to calculate the acoustic speed and thickness in the skull (Hatakeyama et al., 2002). The skull has also been idealized as a shell with non-parallel boundaries, thereby facilitating investigation of the transmission of shear waves in the skull bones by using spectral decomposition (Clement et al., 2004). By considering the irregular surfaces and complex inner structure of the skull, full-wave simulation guided by magnetic resonance imaging and computed tomography (CT) is close to reality (Hayner and Hynynen, 2001; Connor et al., 2002). Different types of full-wave simulation have been used for transcranial ultrasound. The finite-difference time-domain (FDTD) method, which is a conventional full-wave simulation method (Yılmaz and Çiftçi, 2013), has been used to estimate the velocity of longitudinal and shear waves in the human skull (Hughes and Hynynen, 2017). The Fourier pseudospectral time-domain method, utilizing fast Fourier transform to solve acoustic equations, tends to be more efficient in solving large-scale problems (Liu, 1998; Muñoz and Hornikx, 2017). The *k*-space method, which is accurate for weak scattering media, has also been applied in transcranial studies (Mast et al., 2001; Robertson et al., 2017).

However, three-dimensional acoustic full-wave simulations are limited by excessive time consumption and memory requirements (Pichardo et al., 2017). Recently, ray tracing (RT) has been implemented in long-bone structure imaging (Renaud et al., 2018) and phase compensation for B-mode image reconstruction (Szostek and Piórkowski, 2016). It shows potential for wave-path prediction and phase-aberration compensation for transcranial ultrasonic focusing. Commonly used in vision graphics and seismic tomography (Wei et al., 2014), RT is more efficient and requires less computational capability than full-wave simulation. However, the spatially varying porosity of the skull limits the use of RT because the acoustic properties differ spatially even in one ultrasound wavelength, which is beyond the ray regime. Consequently, the skull is generally idealized as being either homogenous or less heterogeneous to satisfy the RT requirements (Jin et al., 2008; Wang and Jing, 2013; Vassilevski et al., 2016). In previous papers, several transcranial ultrasound models have treated the skull as a homogenous medium, for which the ultrasound speed was simplified as the average of the entire skull (Jin et al., 2008; Renaud et al., 2018). However, the validity of that simplification is yet to be discussed.

In the present study, to satisfy the RT requirements, the porosity of the skull bone is simplified and the heterogeneous skull bone is regarded as being homogenous. In turn, for refraction calculation with Snell’s law (SL), the homogeneous properties of the skull bone are simplified as those of either (i) the cortical portion of the skull or (ii) the mean of the entire skull. For each simplification, the transcranial focusing deflections are evaluated and compared with those obtained using the time-reversal method. The paper is organized as follows: in methods section, RT method, *k*-space pseudospectral based full wave-simulation and time-reversal theory are introduced. Then, the numerical implement is introduced, including CT-based heterogeneous assumption of skull bone, homogeneous assumption in RT and simulation setup. In the simulation setup, focusing deflections caused by (i) the shear wave neglection after phase correction, (ii) the presence of skull with conventional focusing algorithm and (iii) homogeneity assumption in RT are investigated. In the discussion section, the focusing deflections of the simulations are given and corresponding discussion is presented. The present investigation of transcranial focusing deflection with RT should (i) improve the understanding of directional wave deflection for ultrasound transmission and (ii) help in choosing optimal acoustic properties to reduce wave-path estimation errors. The present results have meaning for fast and accurate transcranial phase-aberration calculation with RT.

## Materials and Methods

### Ray Tracing for Transcranial Ultrasound

There are two ways to implement RT numerically. The gridded-velocity model, which is based on the Fourier plane-wave assumption, details the velocity field in two or three dimensions; the ray trajectories are then found by solving the geometrical spreading equation $\frac{A}{2}\,\nabla^{2}A-\nabla A\,\nabla T=0$, where *A* and *T* are the amplitude and the travel time functions, respectively, both of which vary with position (Kendall and Thomson, 1989). The alternative model, which assumes multiple layers, specifies the geometrical boundary between different velocity layers and implements SL calculations at the boundary (Waltham, 1988; Clement and Hynynen, 2003). The gridded-velocity model is the simpler of the two models because the RT calculation is reduced to the geometrical spreading equation that incorporates SL; however, computer memory consumption and computational inefficiency impede its use for three-dimensional simulation. The second approach requires complex geometrical calculations for the detailed boundary confirmation and is suitable only in cases of relatively few layers. In the present work, the skull bone is idealized as an isotropic homogenous medium for RT, and a three-layer model is solved by using the latter method for transcranial ultrasonic transmission.

#### Transmission Coefficient

When the spherical wave generated by a point source refracts at a liquid–solid boundary, the ultrasound energy decreases and the amplitude of the velocity potential decreases to approximately (Teng and Zhang, 1997).

(1) $$Q_{2}=\left(Q_{r\,e\,f}\,l_{r\,e\,f}/l_{1}\right)\,T_{r}\,l_{2}^{\prime }/\left(l_{2}^{\prime }+l_{2\,r}\right).$$

Here, we have $l_{2}^{\prime }=l_{1}\,\text{sin}\,\alpha \,{\text{cos}}^{2}\,\beta /\left(\text{sin}\,\beta \,{\text{cos}}^{2}\,\alpha \right)$, where *l*_*1*_ and *l*_*2r*_ are the lengths of the incoming and refracted rays, respectively, α and β are the incoming and refracted angles, respectively, *Q*_*ref*_ is the velocity potential reference, and *l*_*ref*_ is the corresponding distance between the reference and the source. As shown in the Appendix, *T*_*r*_ is the plane-wave transmission coefficient. For the three-layer model, the amplitude of the velocity potential decreases approximately as

(2) $$Q_{p}=Q_{2}^{\prime }\,T\,l_{3}^{\prime }/\left(l_{3}^{\prime }+l_{3}\right).$$

Here, we have $l_{2\,t}=l_{2}+l_{2}^{\prime }$ and $l_{3}^{\prime }=l_{2\,t}\,\text{sin}\,\alpha^{\prime }\,{\text{cos}}^{2}\,\beta^{\prime }/\left(\text{sin}\,\beta^{\prime }\,{\text{cos}}^{2}\,\alpha^{\prime }\right)$, where α′ and β′ are the incoming and refracted angles, respectively, on the second layer, *l*_*2*_ is the ray length between the two layers, *l*_*3*_ is the refracted ray length on the second layer, and $Q_{2}^{\prime }$ is the velocity potential of the incoming ray at the second intersection.

#### Phase Calculation and Waveform Synthesis

Based on the ray shooting method of RT theory, the optimal refraction positions on the two layers can be acquired and the corresponding travel time can be calculated as $t=\sum_{i=1}^{3}\left(l_{i}/c_{i}\right)$, where *c*_*i*_ is the wave speed in layer *i*. The frequency deviation caused by the acoustic attenuation can be calculated in the frequency domain. The distorted waveform and the amplitude of the velocity potential can thus be predicted as *P*(*t*) = ℱ^−1^{ℱ(*P*_*ref*_(*t*))*A*(ω)}, where ℱ and ℱ^−1^ are the forward and inverse Fourier transforms, respectively, and *A*(ω) is the frequency-dependent attenuation coefficient. The ultrasound amplitude after refraction can be acquired with the transmission coefficient as shown in the section entitled “Transmission coefficient.” The multi-reflection at the middle layer can be neglected when the attenuation there is high compared with those at the adjacent layers. The first transmissive waves are considered with the RT method. Because longitudinal and shear waves exist in the middle layer, two rays are derived separately and combined to synthesize the final waveform.

### Full-Wave Simulation

As the theoretical model of ultrasonic propagation, we choose the Kelvin–Voigt elastic wave equation, which includes basic elastic properties such as density, Lamé constants, and attenuation. The corresponding time-domain numerical solutions are acquired using the *k*-space pseudospectral method. In a previous study, various *k*-space algorithms were applied for acoustic wave simulation. The simplest version, which is based on the second-order wave equation, applies to isotropic and homogeneous or weakly homogeneous media (Mast et al., 2001). The second version, which is based on coupled first-order equations, suits sharper-varying materials and requires additional memory to store the displacement vector or the velocity vector (Tabei et al., 2002). Compared with the FDTD method, the second version is more efficient because it requires fewer grid points for the same simulation accuracy. For instance, it is perfectly accurate for homogenous media, even with the two grid points per wavelength that are used in the second version of the *k*-space algorithm, whereas at least six grid points per wavelength are required with the FDTD method (Liu, 1998). The *k*-space pseudospectral algorithm, which is a combination of the two versions, is suitable for large-scale wave simulation because it saves memory and increases computing speed by requiring fewer grids.

#### Kelvin–Voigt Model

In the linear acoustic regime, coupled first-order equations determine the wave propagation in a viscoelastic medium. In temporal differential form, the coupled equations are given as (Carcione et al., 2004).

(3) $$\sigma_{i\,j}\,\left(r,t+\Delta \,t\right)$$

$$=\Delta t\{\lambda \left(r\right)\delta_{i\,j}\sum_{k=1}^{3}\frac{\partial v_{i}\,\left(r,t\right)}{\partial x_{i}}+\mu \left(r\right)\left(\frac{\partial v_{i}\,\left(r,t\right)}{\partial x_{j}}+\frac{\partial v_{j}\,\left(r,t\right)}{\partial x_{i}}\right)$$

$$+\lambda^{{}^{\prime }}\,\left(r\right)\,\delta_{i\,j}\,\sum_{k=1}^{3}\frac{\partial^{2}v_{i}\,\left(r,t\right)}{\partial x_{i}\,\partial t}$$

+μ(r)′(∂2⁡vi⁢(r,t)∂⁡xj⁢∂⁡t+∂2⁡vj⁢(r,t)∂⁡xi⁢∂⁡t)}σi⁢j(r,t),i,j=1,2,3,

(4) $$v_{i}\,\left(r,t+\Delta \,t\right)=\frac{\Delta \,t}{\rho \,\left(r\right)}\,\left(\sum_{j=1}^{3}\frac{\partial \sigma_{i\,j}\,\left(r,t\right)}{\partial x_{j}}+f\,\left(r,t\right)\right)+v_{i}\,\left(r,t\right),i=1,2,3,$$

where σ_*ij*_and*v*_*i*_ are the stress and velocity vectors, respectively,*f* is the external stress, ${\text{δ}}_{ij}\text{⁢}=\text{⁢}\{{}_{0}^{1}\;{\;}_{i\;\neq \;j}^{i\;=\;j}$ is the delta function, and *x*_*i*_ and *x*_*j*_ are spatial directions as *x* = {*x*_1_, *x*_2_, *x*_3_} in Cartesian coordinates. Moreover, ρ(*r*) is the density, λ(*r*) and μ(*r*) are the first and second Lamé constants, respectively, λ(r)′ and μ(r)′ are the attenuation coefficients, and Δ*t* is the temporal differential step. Note that the second-order derivation in Eq. 3 can be simplified to

(5) $$\frac{\partial^{2}v_{i}\,\left(r,t\right)}{\partial x_{j}\,\partial t}=\partial \left(\frac{\partial v_{i}\,\left(r,t\right)}{\partial t}\right)/\partial x_{j}$$

$$=\partial \left\{\frac{1}{\rho \,\left(r\right)}\,\left(\sum_{j=1}^{3}\frac{\partial \sigma_{i\,j}\,\left(r,t\right)}{\partial x_{j}}\right)+f\,\left(r,t\right)\right\}/\partial x_{j},\,i,j=1,2,3.$$

#### *K*-Space Pseudospectral Method

The first-order derivation of variables (σ or *v*) can be obtained by using the *forward* and inverse Fourier transforms of the variables

(6) $$\frac{\partial \left(\cdot \right)}{\partial x_{i}}={ℱ}^{-1}\,\left\{i\,k_{i}\,\text{sinc}\,\left(c_{m}\,k\,\Delta \,t/2\right)\,ℱ\,\left(\cdot \right)\right\},$$

where *c*_*m*_ is the maximum wave velocity and ℱ and ℱ^−1^ are the three-dimensional forward and inverse spatial Fourier transforms, respectively. The operator *ik*_*i*_ is generated from the conventional pseudospectral method. The scalar Green’s function operator sinc(*c*_*m*_*k*Δ*t*/2) is derived from the dyadic Green’s function solution of the second-order elastic wave equation (Liu, 1998). This is an improvement from the pseudospectral method. The elastic wave equations are divided into compressional and shear wave components. The compressional stress matrix $\sigma_{i\,j}^{p}$ and the shear stress matrix $\sigma_{i\,j}^{s}$ are calculated independently in Eq.3, while the total stress σ_*ij*_ in Eq. 4 is the sum of $\sigma_{i\,j}^{p}$ and $\sigma_{i\,j}^{s}$. Although staggered grids are not necessary in this method, they are used to improve the stability and efficiency (Firouzi et al., 2012).

When the shear modulus matrix μ is set to zero, the coupled viscoelastic first-order equations degenerate into the acoustic wave equations for a fluid medium. Under that hypothesis, the stress vector σ in the elastic equations is equivalent to the sound pressure *p* in the fluid medium. During transcranial ultrasonic propagation, the stress vector σ and the sound pressure *p* are continuous at the boundary between solid and liquid, whereas the velocities are continuous at the boundary. Therefore, the viscoelastic model is applicable for simulating ultrasound transcranial transmission.

### Time-Reversal Theory

**FIGURE 1:**
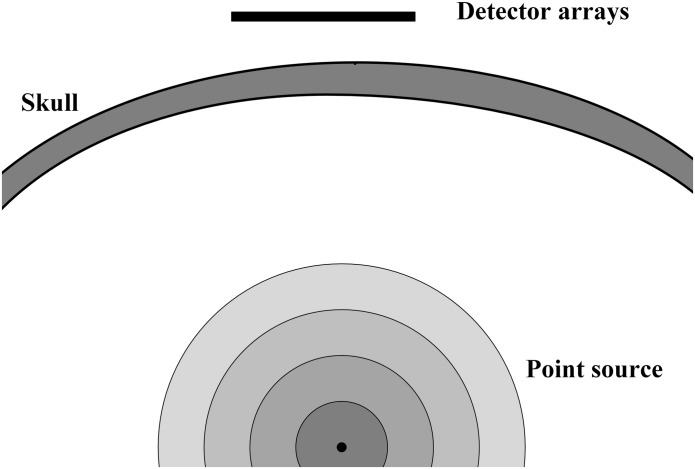
The diagram of VS (point source), skull and detector arrays. VS emits ultrasound wave, which will go through skull and recorded by detector arrays.

For conventional ultrasonic focusing with a phased-array probe, the phase aberrations are based on the assumption of constant sound velocity in soft tissue. However, the wave velocity difference between soft tissue and bone impedes its application in transcranial focusing. Phase correction with time-reversal theory is a valid way to compensate for the distortion that is caused by the skull. Time-reversal theory, which is based on the reciprocity principle, takes advantage of the invariance of the wave equation and assumes that forward and backward ultrasonic propagation have the same time–frequency response. Ultrasound from a virtual or real source that is located at the desired focal point should be recorded by each channel of the phased array (Figure 1). The time-reversed wave that propagates backward to the source will focus optimally on the source (Thomas and Fink, 1996). For conventional time-reversal theory, when the source transmits a pulsed signal, the receivers must record all of the temporal waveforms. The signals are time-reversed and transmitted backward to the source to guarantee the optimal pulse waveform at the focal point. However, transmitting ultrasonic signals with the source deep inside the skull *in vivo* tends to be difficult, especially for clinical trials. An alternative option is to use geometrical information about the skull bone to estimate the waveforms with a virtual source (VS) transmitting a signal inside the skull. For the RT method in the present work, diffraction and multilayer reflections are neglected. Diffractions are omitted because diffraction is weak with the assumption that microstructure (trabecular bone) is not considered and thickness of bone is significantly larger than wavelength. Multilayer reflections are omitted because the energy of reflected waves is neglectable compared with that of the wavefront as a result of attenuation in bone and reflectional energy loss at the tissue-bone boundary. The longitudinal–longitudinal–longitudinal and longitudinal–shear–longitudinal transmission modes are calculated separately and then combined as the signal received by the phased array, while the remaining temporal waveforms are set to zero.

## Numerical Implement

### CT-Based Heterogeneous Plastic Material Properties for *k*-Space Pseudospectral Full-Wave Simulation

**FIGURE 2:**
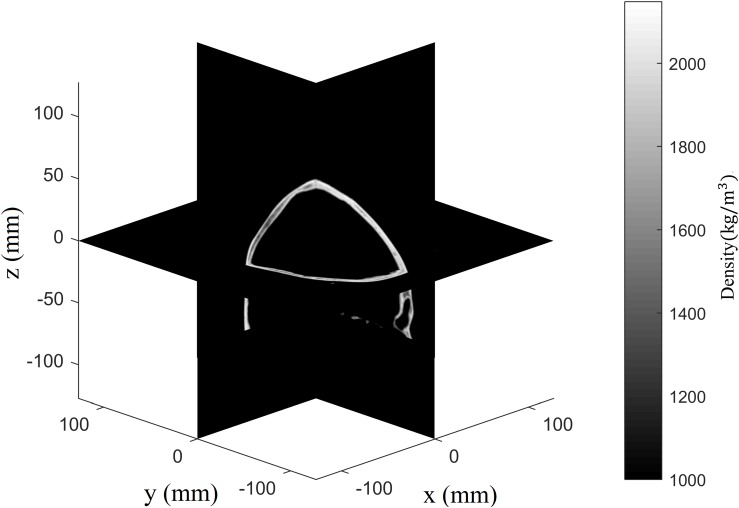
Density distribution of skull and degassed water in vertically spaced slice mode.

In transcranial ultrasonic investigations, the assumption that the skull’s elastic properties vary along with Hounsfield unit in CT-images has been verified experimentally for transcranial focusing (Top et al., 2016; Pichardo et al., 2017). Under that hypothesis, the elastic properties of the computational region, such as density, wave velocity, and attenuation, can be acquired with the following equations (Pichardo et al., 2011, 2017; Top et al., 2016):

(7) $$\psi =1-\frac{H\,u}{H\,u_{w}},$$

$$\rho =\rho_{m\,i\,n}\,\psi +\rho_{m\,a\,x}\,\left(1-\psi \right),$$

$$c_{L}=c_{w}\,\psi +c_{b}\,\left(1-\psi \right),$$

$$\lambda^{\prime }=\lambda_{m\,i\,n}^{\prime }+\left(\lambda_{m\,a\,x}^{\prime }-\lambda_{m\,i\,n}^{\prime }\right)\times \psi^{\beta },$$

where *Hu* is the Hounsfield unit, *Hu*_*w*_ represents the Hounsfield windowing of CT data, ψ is the porosity matrix, which is relevant to the bone trabecular density, and ρ is the density matrix, with ρ_*min*_ = 1000kg/m^3^ as the density of water and ρ_*max*_ = 2100kg/m^3^ as the maximum density of the skull. The density distribution can be acquired for the entire computational region (Figure 2). In addition, *c*_*L*_ is the longitudinal wave speed matrix, with *c*_*w*_ = 1500m/s as the sound speed in water and *c*_*b*_ = 2900m/s as the maximum longitudinal wave speed in the skull. The shear wave speed is approximated as *c*_*s*_ = 7*c*_*l*_/11 in the skull (Pichardo et al., 2017). The term λ′ is the frequency-dependent longitudinal wave attenuation matrix, with $\lambda_{m\,i\,n}^{\prime }=12\,N\,p\,m^{-1}$ as the minimum attenuation coefficient and $\lambda_{m\,a\,x}^{\prime }=460\,N\,p\,m^{-1}$ as the maximum attenuation coefficient (Pichardo et al., 2011). The shear wave attenuation matrix is set to 20λ/′19 (Top et al., 2016), with β = 0.5.

### Homogeneous Models for Ray Tracing

In the idealization of using conventional homogeneous elastic properties for transcranial focusing and imaging, a uniform velocity has been treated as being the average for the entire skull, whereas the focusing accuracy remains to be evaluated (Tretbar et al., 2009). However, we consider the velocity in the cortical bone as being superior to an average velocity, this being because the cortical bone covers the skull and refraction occurs at the boundary. We investigate the accuracy of the two idealizations. For the first case, the constant ultrasonic longitudinal velocity in the skull layer is taken as *c*_*al*_ = 2358m/s and shear velocity is taken as *c*_*as*_ = 1500m/s, which is the average velocity of the skull. The constant density is taken as the average value, namely ρ = 1656kg/m^3^. For the second case, the velocity on the surface of the skull layer is taken to be that in the cortical bone, namely *c*_*cl*_ = 2900m/s and shear velocity is taken as *c*_*cs*_ = 1845m/s. The density is taken as being the maximum density, namely ρ = 2100kg/m^3^, which is used for the SL-based refraction calculation. The internal skull velocity is taken as being the average value on the ray paths, namely *c* = 2358m/s, which influences the travel time in the skull layer.

### Simulation Setup

Because the acoustic properties of soft tissue, such as the scalp, cerebral spinal fluid, and intracranial soft tissues, are comparable with those of water, all the soft tissues are treated as water. An *in vitro* skull is assumed to be immersed in degassed water to avoid the adverse effects of bubbles, such as acoustic scattering, energy attenuation, and non-linearity. The pixel interval for the whole computational region is interpolated to be 0.5 mm to meet the minimum demand of full-wave simulation, that the mesh size (pixel interval) is approximately one-fourth the wavelength λ = 1.93 mm in water. The corresponding grid size is 512 × 512 × 512, with the skull placed in the central region. With a central frequency of 0.8 MHz and an active element spacing of 10 mm for transcranial focusing, the planar phased array is located 5 mm above the upper surface of the skull and comprises 10 × 10 elements. Although the relatively large element spacing leads to grating lobes, it does not interfere with the main lobe, which is the present emphasis. The default VS is located at the center of the grid of the computation region, which is also the origin of the rectangular coordinate system. In addition, the axial line of the entire computation region, namely the *z* axis of the rectangular coordinate system, runs perpendicularly through the middle of the planar phased array. However, we do not consider the size and direction sensitivity of each element or the bandwidth of the phase array (Hu et al., 1988).

The *k*-space pseudospectral method based on the elastic model tends to be superior to the conventional fluid model because neglecting shear waves in the latter influences the transcranial ultrasonic focusing position and intensity even when the incoming incident wave does not exceed the critical angle for shear-wave omission. The transcranial propagations in this section are calculated with the CT-based heterogeneous-medium assumption, and simulations are implemented to evaluate the impact of neglecting shear waves. In the first case, longitudinal and shear waves in the skull are considered both for the VS to array receiver (VS2AR) process and the array receiver to VS (AR2VS) process. In the second case, longitudinal and shear waves in the skull are considered for the VS2AR process, whereas the shear waves are neglected for the AR2VS process. The neglecting of shear waves is discussed in this section only; in all other sections, longitudinal and shear waves are considered by default for wave-propagation simulations. Note also that in all other sections, transcranial propagation is calculated with the heterogeneous-medium assumption using the elastic model based on the *k*-space pseudospectral method for the AR2VS process.

Focusing zone deflection of transcranial ultrasound has been investigated with spherically focusing phased array (Hughes and Hynynen, 2017). However, deflection with planar phase array remains to be investigated. Thus, simulations are implemented to evaluate the tremendous impact on focusing zone of skull-induced distortion. Firstly, temporal waveforms are derived with the conventional focusing algorithm without considering the skull’s presence for the VS2AR process. The waveforms are time-reversed and transmitted backward toward the VS. Secondly, the skull is not considered for the AR2VS process in the first case but is located between the transducer array and the VS in the complementary case.

In order to evaluate the influence of middle layer (cancellous bone) on focusing zone deflection. Simulations were implemented where temporal waveforms are derived with different homogenous idealizations by using the RT method for the VS2AR process. Re-focusing the deviations with the two homogenous idealizations, the mean velocity value of the entire skull and of the cortical bone are evaluated separately, as mentioned previously. The purpose is to investigate the optimal choice for efficient and accurate RT-based transcranial focusing. Groups of simulations are implemented with VSs other than the default one. The focusing deviations, whose temporal waveforms are calculated by using RT when the homogenous properties of the skull are idealized as those of the cortical bone for the AR2VS process, are measured and compared with those of the conventional time-reversal method. When the temporal waveforms are derived with RT, the time-reversed signal that is emitted from each array element is normalized according to the channel with the highest intensity. The waveform is also set as that for the channel with the highest intensity. This normalization makes sense because the present concern is the focusing deviation, not the power.

## Results

### Accuracy and Calculation Efficiency

**FIGURE 3:**
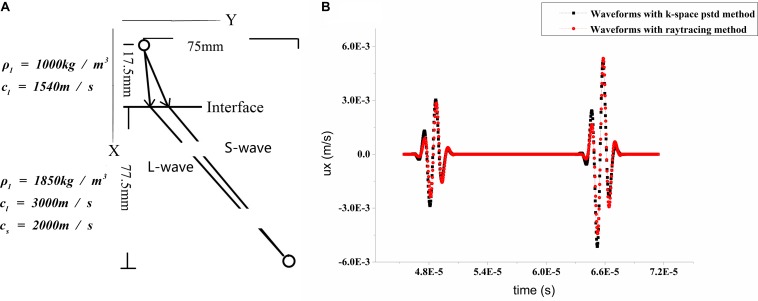
**(A)** Diagram of ultrasonic refraction at liquid–solid boundary. **(B)** Corresponding waveforms of velocity in *x* direction with *k*-space pseudospectral method (black) and ray tracing (RT) (red). The first pulse is caused by a longitudinal wave and the second pulse is caused by a shear wave.

The *k*-space pseudospectral method, the pseudospectral method, and the FDTD method are compared to evaluate the accuracy of the full-wave simulation. The waveform with the *k*-space pseudospectral method (λ = 4Δ*x*, *u*_*max*_Δ*t*/Δ*x* = 0.1) has a phase-error ratio of 0.7% compared with that with the FDTD method (λ = 16Δ*x*, *u*_*max*_Δ*t*/Δ*x* = 0.015), where λ is the wavelength, Δ*x* is the grid width, Δ*t* is the time interval. Phase-error ratio is represented by Δφ/2π×100%, where Δφ is the phase difference. The waveform with the pseudospectral method has a phase-error ratio of 7.3% compared with that with the FDTD method under the same setting. The *k*-space pseudospectral method is suitable for the present simulation as it has better accuracy under the same sparse spatial and temporal grids compared with the pseudospectral method. After refraction at the liquid–solid boundary, the waveforms of the acoustic velocity are calculated using RT and the *k*-space pseudospectral method separately (Figure 3). The results confirm the feasibility of RT with an amplitude error $\left(\frac{A}{A_{F\,D\,T\,D}}-1\right)\times 100\%$ of 5.35% and a phase-error ratio of 1.2%, where *A* is the amplitude with *k*-space pseudospectral method or with pseudospectral method and *A*_*FDTD*_ is the reference amplitude with FDTD. The computational time is reduced from 23 h 35 min 13 s with the *k*-space pseudospectral method to 37 min 24 s with RT (Intel^®^ Xeon^®^ E7-4830 v4; MATLAB 2017; 12 cores for parallel computation).

### Focusing Deviation Caused by Omission of Shear Waves

**FIGURE 4:**
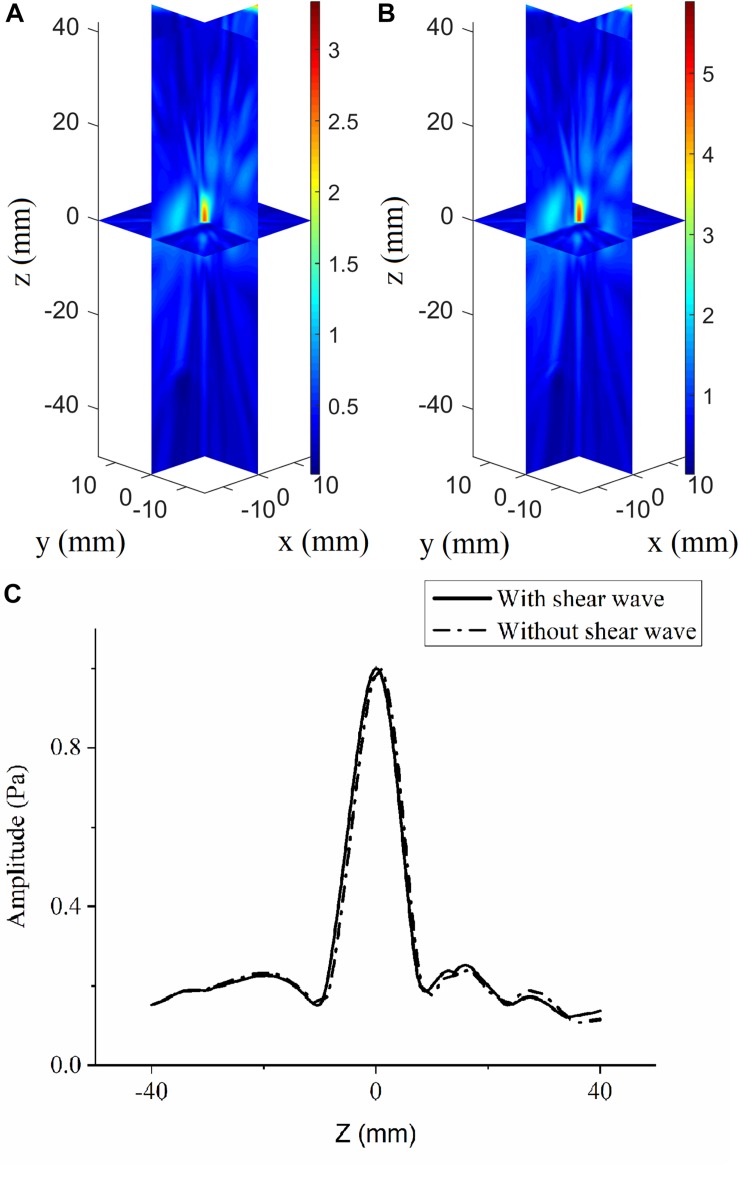
Three-dimensional pressure distributions using conventional time-reversal method considering **(A)** and without considering **(B)** the shear wave for the virtual source to array receiver (VS2AR) process. **(C)** The corresponding normalized pressure distribution in the axial direction.

The angles between the incident incoming waves from the array elements to the VS and skull surfaces are less than 20°, which meets the demand of shear-wave omission. The acoustic pressure distributions are illustrated in vertically spaced slice mode, and the deviations in focal position are small compared with the large ultrasonic field space when shear waves are considered (Figure 4A) and when they are not (Figure 4B). The maximum pressure in the focusing area when shear waves are considered is approximately 3.65 Pa, while that when shear waves are not considered is approximately 4.85 Pa. The normalized pressure distributions in the axial direction are illustrated for better distinction (Figure 4C); they reveal an overall distortion of 0.5 mm beyond the VS when shear waves are not considered and perfection at the VS when shear waves are considered.

### Presence of Skull-Induced Focusing Error With Conventional Phased Array Focusing

**FIGURE 5:**
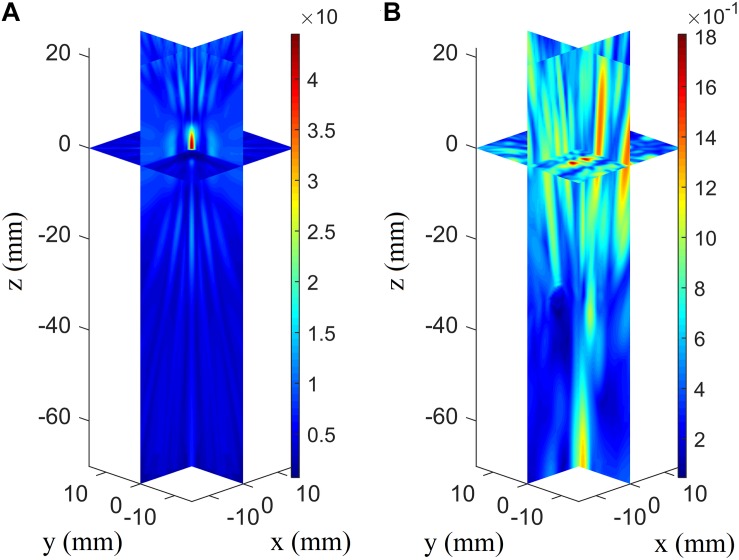
Three-dimensional pressure distributions using conventional focusing algorithm without the skull’s presence **(A)** and with the skull’s presence for the array receiver to virtual source (AR2VS) process **(B)**.

When evaluating the tremendous impact of skull-induced distortion, the focusing deviations are illustrated better by showing them with their coordinates moving up in the axial direction (Figure 5). The phased array achieves optimal focusing using the conventional phased-array focusing algorithm without the presence of the skull, while the focusing position with the presence of the skull shows deflections of 79.0 mm in the axial direction and 3.5 mm in the focal plane. For conventional focusing without the skull, the maximum pressure in the focusing area is approximately 46.5 Pa, whereas that with the skull is approximately 1.95 Pa. The pressure in the focusing area is low because the ultrasound transmitted from phased array are normalized according to the maximum. The phenomenon of focusing-area elongation compared with the ideal focusing zone is attributed to the acoustic field of the phased array, which elongates the main lobe with increasing focusing depth. Note that the attenuation disparity leads to the difference in pressure amplitude between the two cases.

### Focusing Deviation After Phase-Aberration Correction With Ray Tracing

**FIGURE 6:**
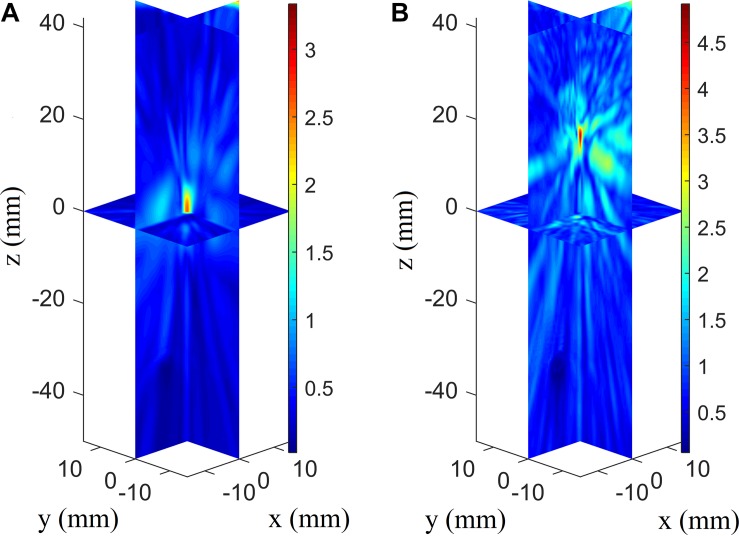
Three-dimensional pressure distributions with temporal waveforms derived using RT when the homogenous properties of the skull are simplified as those of the cortical bone **(A)** and the whole-skull average **(B)** for refraction calculation for the VS2AR process.

**FIGURE 7:**
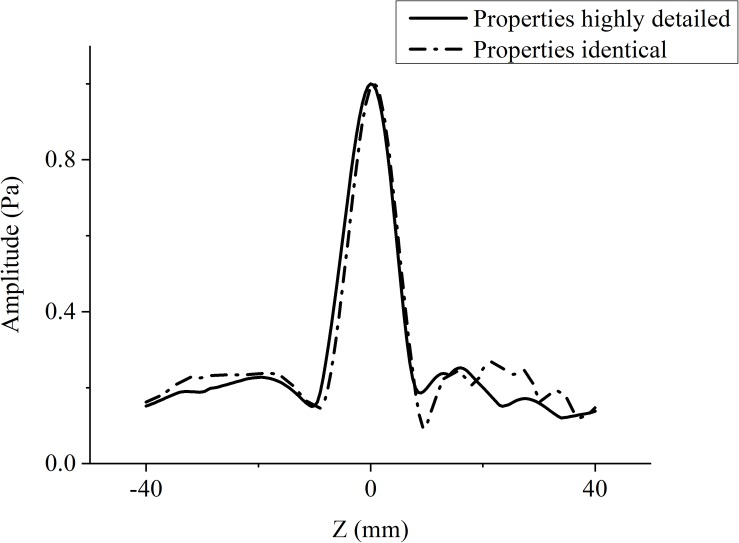
Normalized pressure distribution in axial direction when temporal waveforms are calculated using RT with homogenous properties of the skull idealized as those of the cortical bone for refraction calculation (broken line), and the conventional time-reversal method (solid line) for the VS2AR process.

**FIGURE 8:**
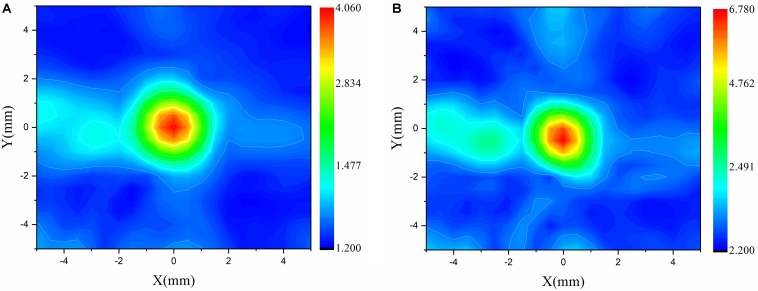
Normalized pressure distribution in focal plane when temporal waveforms are calculated using the conventional time-reversal method **(A)** and RT with the homogenous properties of the skull idealized as those of the cortical bone for refraction calculation **(B)** for the VS2AR process.

When the temporal waveforms are derived with RT under the assumption that the homogenous properties of the skull are simplified as those of the cortical bone for refraction calculation, the focusing distribution reveals deflections of 0.5 mm in the axial direction and 0.5 mm in the focal plane compared with the VS (Figure 6A). By contrast, the focusing distribution reveals deflections of 9.5 mm in the axial direction and 1.5 mm in the focal plane when the homogenous properties of the skull layer are simplified as those of the mean of the entire skull (Figure 6B). The focusing deflections are evaluated when the homogenous properties of the skull are those of the cortical bone for refraction calculation with RT (Figure 6A) and are compared with those of the conventional time-reversal method (Figure 4A). The normalized pressures are extracted and reveal a quasi-Gaussian distribution with a main-lobe width of 11 mm in the axial direction (Figure 7) and a two-dimensional quasi-Gaussian distribution with a main-lobe width of 1.5 mm in the focal plane (Figure 8). The focusing deviations, including source position, spatial deviation, main-lobe width, and deviation ratio in each direction, are given in Table 1 to illustrate the influence of homogeneous idealization using RT. The deviation ratio is the result of dividing the spatial deviation by the main-lobe width.

**TABLE 1:** Focusing deviations with different VSs (mm).

	Deviation in each direction		
Virtual source	*x*	*Y*	*z*
(0.0, 0.0, 0.0)	(1.0, 4.0, 25)	(0, 4.0, 0)	(0.5, 16.0, 3.0)
(10.0, 0.0, −10.0)	(0.5, 4.0, 12.5)	(0, 4.5, 0)	(0.5, 17.0, 3.0)
(−10.0, 0.0, −10.0)	(0, 3.5, 0)	(0, 4.5, 0)	(0.5, 16.5, 6.7)
(0.0, 0.0, −10.0)	(0, 3.0, 0)	(0, 3.5, 0)	(0.5, 16.5, 3.1)
(10.0, 10.0, 0.0)	(1.0, 3.5, 14.3)	(1.0, 4.0, 12.5)	(0.5, 14.5, 3.5)
(10.0, −10.0, 0.0)	(1.0, 3.5, 14.3)	(1.0, 4.0, 12.5)	(0.5, 14.5, 3.5)

*Format of deviations in each direction is as follows: focusing-position deviation (mm), main-lobe width (mm), deviation ratio (%).*

## Discussion

For the AR2VS process, the ultrasonic pressure and focusing position differ slightly depending on whether shear waves are considered. To some extent, the results show that shear waves can be neglected in less-rigorous cases in which the incident wave does not exceed the critical angle for shear-wave omission. However, neglecting the shear waves influences the focusing accuracy and is better when using the elastic model rather than the fluid model. The deviations can be interpreted as the fact that the longitudinal wave in the skull plays a major role as that of the small incident wave, and a small portion of ultrasound energy in fluid medium is transformed into shear waves in solid medium during longitudinal–shear–longitudinal transmission. Different velocities of longitudinal and shear waves in the skull lead to different refractions and wave paths if the longitudinal–shear–longitudinal and longitudinal–longitudinal–longitudinal transmission models are considered separately. In the present work, the focusing position of the longitudinal–shear–longitudinal model happens to be lower than that of the VS, while the focusing position of the longitudinal–longitudinal–longitudinal model is higher than that of the VS. The two focuses are mixed to form the VS. So, the focusing position is a little above the VS if shear wave in bone is not considered. In addition, the maximum amplitude of the focus region with shear waves considered is smaller than that with shear waves neglected, this being because shear waves are attenuated more than are longitudinal waves. It is predictable that this phenomenon should become more obvious as the VS moves closer to the skull, which is equivalent to increasing the angle of the incident wave. The extensive applicability of the elastic model shows its advantages in transcranial investigation, especially for rigorous circumstances. Certain studies have discussed how neglecting shear waves influences transcranial investigations. For example, neglecting the refraction and mode conversion of shear waves in the skull layer for transcranial ultrasonic imaging has led to the images of the absorbers being blurred and dislocated; such phenomena become more evident as the absorbers move closer to the skull both in simulations and in experiments (Jin et al., 2008). Also, the effects of shear-wave propagation in three layer models have been investigated to estimate the compensation of Fourier components in plane-wave representation for image reconstructions using photoacoustic tomography (Schoonover et al., 2012). In conclusion, the elastic model based on the *k*-space pseudospectral method is superior in both computational efficiency and accuracy and is optimized for the transcranial ultrasonic scenario.

The illustrations in Figure 5 reveal the distinct influence of skull-induced distortion on transcranial focusing. The high attenuation of the skull bone is attributed to the marked difference in the focusing amplitude. In addition, the focusing deviations are large—especially in the axial direction—compared with the results of some studies on transcranial focusing therapy (Kyriakou et al., 2013). That is because focused array transducers are generally used for ultrasonic therapy, and the corresponding distortion was not intense, especially when the VS was not far from its self-focus point (Kyriakou et al., 2013). In ultrasound imaging, using the conventional delay-and-sum reconstruction algorithm for transcranial imaging is expected to give either erroneous or distorted images of brain tissue. Wang and Jing (2013) discussed transcranial imaging with skull aberration, where the positions rising of wire phantom images in the axial direction considering the skull without phase correction is caused by a sound-speed mismatch between skull and tissue. The shape disorder and image deflection in the radial direction are caused by the deflection of the focusing position in the radial plane. There are some unexpected wire phantom artifacts that can be interpreted as the influence of side-focuses (or sidelobes) from skull-induced ultrasonic distortion (Figure 5B). The related studies indicate that phase-correction algorithms are required to solve the skull-induced distortion.

Comparing the focusing deviations indicates that the velocity value of cortical bone, instead of the average velocity value, is more suitable for temporal waveform estimation with RT for refraction calculation. The reason is that the skull comprises three layers, namely (i) the upper cortical layer, (ii) the cancellous layer, and (iii) the lower cortical layer. The cortical layer, which is the hardest portion of the skull, is likely to play a decisive role in ultrasonic refraction at the skull–liquid boundary. Cancellous bone, which has a trabecular structure, will not change the ultrasonic propagation path significantly. In this idealization, the fine structure of cancellous bone (heterogenous medium) is simplified as homogenous medium, where refraction-induced ray-path deflections inside the skull are neglected. The focusing deviations indicate that heterogeneity inside the skull has limited influence on the ultrasonic path deflection. The focusing deviation result also supplements the discussion of how the fine structure influences phase aberration, namely that the phase of the ultrasound rarely changes even if the fine structure in the skull is down sampled to half-wavelength resolution (Jing et al., 2012). To investigate the feasibility of the homogenous idealization with RT, deviations with diverse VSs were examined. The focal positions and widths were always integers in multiples of 0.5 mm, as a result of the fixed spatial resolution and the locations. The deviation lengths for different VSs are random and less than 1 mm, revealing average deviation ratios of 8.6% in the *x* direction, 3.9% in the *y* direction, and 3.2% in the *z* direction. The deviation ratios in the focal plane are higher than their counterparts on the axial line because the semi major axis of the ellipsoidal focal area lies in the axial direction. The deviations reveal the reliability of using RT to estimate the temporal waveforms when the skull surfaces are idealized as cortical bone for refraction calculation. The present results contribute to the analysis of unpredictable bending of wave trajectories caused by the trabecular layer and thus provide further insight into major and minor factors of transcranial wave directional deflection, which can be meaningful for fast and accurate phase-aberration correction calculation.

## Conclusion

Transcranial focusing deviations are evaluated when the phase aberrations are corrected with RT. The homogenous properties of the skull are idealized as those of either the cortical bone or the average of the whole skull. The results reveal that the cortical bone, instead of the average of the whole skull, should be used for homogenous idealization with RT. The deviations also indicate that the heterogeneity inside the skull bone plays a marginal role in transcranial aberration, which can be neglected if precise calculation is not demanded. The transcranial ultrasonic transmission process was implemented with the Kelvin–Voigt viscoelastic model using the *k*-space pseudospectral method, where longitudinal waves, shear waves, and attenuation are all considered. The model shows extensive applicability and accuracy compared to the regularly used fluid model, offering guaranteed reliability of transcranial investigation. The present results could help with estimating wave paths for fast and accurate phase correction using RT, which contribute to application of transcranial ultrasound in brain computer interface systems. Our future work will focus on the *in vivo* experiments of transcranial ultrasound focusing and neuromodulation with the focused ultrasound.

## Data Availability Statement

The datasets generated for this study are available on request to the corresponding author.

## Author Contributions

CJ and DT conceived the idea of the study. CJ, DL, FX, YL, and CL analyzed the data, interpreted the results and wrote the manuscript. All authors discussed the results and revised the manuscript.

## Conflict of Interest

The authors declare that the research was conducted in the absence of any commercial or financial relationships that could be construed as a potential conflict of interest.

## Appendix

According to the Zoeppritz equations (Shuey, 1985), the plane-wave transmission coefficients can be derived for the liquid–solid boundary. A longitudinal wave in the liquid material will transform into shear and longitudinal waves in the solid material. The reflection and transmission coefficients are presented as

$$\left(\begin{array}{ccc} A_{1}\; & B_{1}\; & C_{1}\\ A_{2}\; & B_{2}\; & C_{2}\\ A_{3}\; & B_{3}\; & C_{3} \end{array}\right)\,\left(\begin{array}{c} R_{l\,l}\\ T_{l\,l}\\ T_{l\,s} \end{array}\right)=\left(\begin{array}{c} D_{1}\\ D_{2}\\ D_{3} \end{array}\right),$$

where $A_{1}=-1,B_{1}=\rho_{2}/\rho_{1}\,\text{cos}\,\left(2\,\theta_{t\,s}\right),C_{1}=-\rho_{2}/\rho_{1}\,\text{cos}\,\left(2\,\theta_{t\,s}\right),D_{1}=1;A_{2}=0,B_{2}=\text{sin}\,\left(2\,\theta_{t\,l}\right)/c_{2\,l}^{2},C_{2}=\text{cos}\,\left(2\,\theta_{t\,s}\right)/c_{2\,t}^{2},D_{2}=0;$*A*_3_ = cos(θ_*i*_)/*c*_1*l*_, *B*_3_ = cos(θ_*t**l*_)/*c*_2*l*_, *C*_3_ = −sin(θ_*t**s*_)/*c*_2*s*_, *D*_3_ = cos(θ_*i*_)/*c*_1*l*_, and *R*_*ll*_, *T*_*ll*_, and *T*_*ls*_ are the longitudinal-wave reflection coefficient, longitudinal-wave refraction coefficient, and shear-wave refraction coefficient, respectively. Here, ρ_*1*_ and *c*_*1l*_ are the density and longitudinal wave speed, respectively, of the liquid material, ρ_*2*_, *c*_*2l*_, and *c*_*2s*_ are the density, longitudinal wave speed, and shear wave speed, respectively, of the solid material, and θ_*i*_, θ_*tl*_, and θ_*ts*_ are the angles of the incoming wave, refracted longitudinal wave, and refracted shear wave, respectively.

The longitudinal wave transforms into longitudinal and shear waves after reflection in the solid material while remaining a longitudinal wave in the liquid material. The reflection and transmission coefficients are presented as

$$\left(\begin{array}{ccc} A_{1}\; & B_{1}\; & C_{1}\\ A_{2}\; & B_{2}\; & C_{2}\\ A_{3}\; & B_{3}\; & C_{3} \end{array}\right)\,\left(\begin{array}{c} R_{l\,l}\\ R_{l\,s}\\ T_{l\,l} \end{array}\right)=\left(\begin{array}{c} D_{1}\\ D_{2}\\ D_{3} \end{array}\right),$$

where *A*_1_ = *c**o**s*(θ_*i*_)/*c*_1*l*_, *B*_1_ = −sin(θ_*r**s*_)/*c*_1*s*_, *C*_1_ = cos(θ_*t**l*_)/*c*_2_, *D*_1_ = cos(θ_*i*_)/*c*_1*l*_; *A*_2_ = −*cos*⁡(2θ_*r**s*_), *B*_2_ = sin(2θ_*r**s*_), *C*_2_ = ρ_2_/ρ_1_,$D_{2}=\cos\left(2\,\theta_{r\,s}\right);A_{3}=c_{1\,s}^{2}/c_{1\,l}^{2}\,{\text{sin}}^{2}\,\left(\theta_{i}\right),$*B*_3_ = cos(2θ_*r**s*_), *C*_3_ = 0, $D_{3}=c_{1\,s}^{2}/c_{1\,l}^{2}\,{\text{sin}}^{2}\,\left(\theta_{i}\right)$, and *R*_*ll*_, *R*_*ls*_, and *T*_*ll*_ are the longitudinal-wave reflection coefficient, shear-wave reflection coefficient, and longitudinal-wave refraction coefficient, respectively. Here, ρ_*1*_, *c*_*1l*_, and *c*_*1s*_ are the density, longitudinal wave speed, and shear wave speed, respectively, of the solid material, ρ_*2*_ and *c*_*2l*_ are the density and longitudinal wave speed, respectively, of the liquid material, and θ_*i*_, θ_*tl*_, and θ_*rs*_ are the angles of the incoming wave, the refracted longitudinal wave, and the reflected shear wave, respectively.

e shear wave transforms into longitudinal and shear waves after reflection in the solid material and transform into a longitudinal wave in the liquid material. The reflection and transmission coefficients are presented as

$$\left(\begin{array}{ccc} A_{1} & B_{1} & C_{1}\\ A_{2} & B_{2} & C_{2}\\ A_{3} & B_{3} & C_{3} \end{array}\right)\,\left(\begin{array}{c} R_{s\,s}\\ R_{s\,l}\\ T_{s\,l} \end{array}\right)=\left(\begin{array}{c} D_{1}\\ D_{2}\\ D_{3} \end{array}\right),$$

where *A*_1_ = −sin(θ_*i*_)/*c*_1*s*_, *B*_1_ = −cos(θ_*r**l*_)/*c*_1*l*_, *C*_1_ = cos(θ_*t**l*_)/*c*_2_, *D*_1_ = sin(θ_*i*_)/*c*_1*s*_; *A*_2_ = 0, *B*_2_ = ρ_1_cos(2θ_*i*_), *C*_2_ = ρ_2_, *D*_2_ = 0;$A_{3}=-\text{cos}\,\left(2\,\theta_{i}\right),B_{3}=\text{sin}\,\left(2\,\theta_{r\,l}\right)\,c_{1\,s}^{2}/c_{1\,l}^{2},C_{3}=0,D_{3}=\text{cos}\,\left(2\,\theta_{i}\right)$, and *R*_*ll*_., *R*_*ls*_, and *T*_*ll*_ are the longitudinal-wave reflection coefficient, shear-wave reflection coefficient, and longitudinal-wave refraction coefficient, respectively. Here, ρ_*1*_, *c*_*1l*_, and *c*_*1s*_ are the density, longitudinal wave speed, and shear wave speed, respectively, of the solid material, ρ_*2*_ and *c*_*2l*_ are the density and longitudinal wave speed, respectively, of the, and θ_*i*_, θ_*tl*_, and θ_*rs*_ are the angles of the incoming wave, the refracted longitudinal wave, and the reflected shear wave, respectively.
